# Trends in internal migration in Japan, 2012–2020: The impact of the COVID‐19 pandemic

**DOI:** 10.1002/psp.2634

**Published:** 2022-11-20

**Authors:** Masaki Kotsubo, Tomoki Nakaya

**Affiliations:** ^1^ Graduate School of Environmental Studies Tohoku University Sendai Japan

**Keywords:** COVID‐19 pandemic, internal migration, Japan, migration efficiency

## Abstract

In the past 10 years or so, there have been growing concerns in Japan that migration trends, such as large in‐migration to the Tokyo metropolitan area (TMA) at the national scale and large movements from the suburbs to the centres at the metropolitan scale, have widened the core–periphery disparities at each scale under national population decline. However, the COVID‐19 pandemic led to unexpected changes in these migration patterns, such as a weakened population concentration in the TMA. This study aims to examine internal migration trends from 2012, on axes of core–periphery in Japan and centre–suburbs in metropolitan areas, and the changes in the migration patterns caused by the pandemic in 2020. First, we prepared intermunicipal origin–destination tables by estimating the suppressed flows in 2012–2020 using the iterative proportional fitting technique, and then we calculated the net migration and migration efficiency indices based on seven area types: centre and suburbs of the TMA, centres and suburbs of major metropolitan areas, centres and suburbs of other metropolitan areas and nonmetropolitan areas. The results indicate that the 2020 migration efficiency in the centre of the TMA drastically dropped to the lowest level during the study period, contrasting with an upward trend in 2012–2019. The pandemic strongly affected the migration patterns from/to and within the TMA, with much net gain in the suburbs from the centre, but the impact of migration efficiencies among the other areas was minimal. These findings can help in providing direction for the postpandemic policy challenge of spatial planning in Japan, for example, the weakened but still widening disparities between the TMA and the other regions and the renewed threat of urban sprawl caused by the increased migration from the centre to the suburbs within the TMA.

## 1 INTRODUCTION

Internal migration is one of the major factors responsible for changes in regional population distribution and composition within a country. In Japan, numerous migration studies have been conducted over the past dozen years or so, ever since the national population decline began in 2008 (Ishikawa, 2020a), reflecting the increased attention to internal migration effects in both academic and policy arenas. Regional disparities have also been observed in population change: many municipalities located in provincial Japan have seen an enormous population decline, while a small number of large cities have seen net gains (Morikawa, 2020), particularly the Tokyo metropolitan area (TMA), which has seen large in‐migration (Fielding, 2016, 2018; Ishikawa, 2020b).

The main reasons for large cities witnessing population gains, especially the TMA, are the abundant employment opportunities they offer and the income differences between these areas and the provinces (Ishikawa, 2020b). Most of Japan's advanced developments in technology are centralised in the TMA. Moreover, since the 2000s, information services have boomed, and various service industries make up a significant proportion of the industry in the TMA (Matsubara, 2014). Owing to these regional disparities, the TMA has been the hub of upward socio‐occupational mobility (Fielding, 2018; S. Nakagawa, 2000) and the preferred destination for well‐educated young people from other parts of the country (Takami, 2018).

There have been growing concerns that this migration trend would lead to the disappearance of quite a few rural municipalities (Masuda, 2014); to counter this, the Japanese government established a regional revitalisation policy in 2014 (Morikawa, 2020). With the collapse of the ‘bubble’ economy in the early 1990s leading to a steep decline in land prices in the central parts of metropolitan areas, a large movement of population was seen in the latter half of the 1990s towards the central parts of cities (Inoue et al., 2022). These changes led to a decrease in the number of households and an increase in vacant housing in suburban areas, signifying the controversial issue of shrinking cities in Japan (Yamagami, 2013).

Trends in internal migration throughout the world have been drawing attention in the aftermath of the COVID‐19 pandemic. Fielding and Ishikawa (2021) reported a weakened population concentration in the TMA. They also showed changes in the destinations of migrants from Tokyo based on interprefectural migration flows. The reported destinations in the movement from Tokyo are less frequently prefectures adjacent to Tokyo and increasingly prefectures in provincial areas distant from Tokyo. In addition, the Cabinet Office (2021) reported an increase in the share of people living in the TMA developing a keen interest in residential relocation to provincial areas when compared to the trend in December 2019. While government policies cannot mitigate population concentration in the TMA (Saito, 2021), the changes wrought by the pandemic led to an unintended mitigation of the long‐term trend of ‘mono‐polar concentration into Tokyo’ (Fielding & Ishikawa, 2021; Inoue et al., 2022).

Changes in internal migration patterns during the COVID‐19 pandemic, such as massive out‐migration from urban areas, were reported in other countries as well (Borsellino et al., 2022; González‐Leonardo et al., 2022; Stawarz et al., 2022; Tønnessen, 2021). It has been suggested that the motives behind migration during the pandemic have been related to the changes in people's daily lives (Haslag & Weagley, 2021). Moreover, previous studies have highlighted the increased move towards suburban areas caused by the widespread adoption of telework as a key response to the pandemic (de Abreu, 2022; Denham, 2021). On the same lines, viewing recent migration patterns in policy terms (Denham, 2021) requires grasping the trends of internal migration by considering the distinction between the centre and the suburbs of metropolitan areas.

Although the trends in internal migration in Japan have been discussed at the 47‐prefecture scale (e.g., Fielding & Ishikawa, 2021; Hauer et al., 2020), mainly due to current data limitations, prefectures are not necessarily appropriate to examine migration trends when considering core–periphery regional distinctions. For example, the TMA comprises four prefectures, Tokyo, Saitama, Chiba, and Kanagawa, but these prefectures include municipalities that are part of both the centre and suburbs of the TMA, as well as nonmetropolitan/rural areas.

To overcome this issue, we use the recently upgraded migration data for Japan. The statistics, including origin–destination (OD) matrices of annual intermunicipal migration, have been made publicly available since 2012. A municipality is a finer geographic administrative unit in Japan compared to a prefecture; Japan's municipalities numbered 1896 in 2020. These data allow us to observe temporal changes in internal migration patterns with area classifications using this finer geographical unit. However, in the published OD matrices, cells with small migrant numbers are subjected to suppression, thus obscuring accurate flow counts.

In this study, we aim to examine the trends in internal migration in Japan using annual intermunicipal migration flows. To overcome the suppression in the OD matrices, we estimate the suppressed number of migrants in the OD matrices by using the iterative proportional fitting procedure. We then investigate macrotrends in internal migration in Japan focusing on net migration and migration efficiency based on the area classification that distinguishes the TMA, other metropolitan areas and nonmetropolitan areas, as well as the centre and suburbs of metropolitan areas. One of our main interests in this paper is to investigate the impact of the COVID‐19 pandemic on internal migration patterns, with distinctions of core–periphery in Japan and centre–suburbs in metropolitan areas. To examine the impact of the COVID‐19 pandemic, it is necessary to grasp trends in internal migration in Japan before the pandemic. Therefore, we compare and discuss trends in internal migration in 2012–2019 and in 2020.

The remainder of this paper is organised as follows. The next section summarises the situation of the COVID‐19 pandemic in Japan and previous studies of internal migration and migration intentions during the pandemic in Japan. This is followed by a description of the method of estimating suppressed intermunicipal migration flow in Japan based on the details in the available data sets. This section includes details of the area classification and measurement of changes in migration patterns we used. The section following this presents the results, and the final section is the conclusion, discussing the implications and limitations of the study. We list all abbreviations and acronyms used in this paper in  Table A1.

## 2 BACKGROUND

In Japan, the first confirmed case of COVID‐19 infection was on 16 January 2020 (Ministry of Health, Labour and Welfare, 2020), and the number of confirmed cases increased from there. In April 2020, a state of emergency was first implemented to reduce human‐to‐human contact to prevent the spread of infection considering the rise in the number of COVID‐19 cases. The central part of the TMA experienced the most serious outbreaks, resulting in a prominent decrease in outings in the region (Hanibuchi et al., 2021; Nagata et al., 2021). Due to the high density of the population, high dependency on public transportation and limited large open spaces in the central part of the TMA, outings were impossible without human‐to‐human contact. Under such conditions, teleworking became the norm in the TMA, as to a lesser extent elsewhere in Japan. According to a survey conducted by the Cabinet Office, the rate of teleworking in Tokyo's special wards increased from 17.8% in December 2019 to 42.8% in December 2020 (Cabinet Office, 2021). However, Tokyo's relatively small housing spaces are not suitable for staying home for a long time (Scarr et al., 2020), and this situation thus led to increased mental health problems among the residents during the pandemic (Okubo et al., 2021; Teng et al., 2022).

The above situational changes may have caused changes in internal migration patterns and intentions in Japan. Previous studies have identified some rural areas outside the TMA as destinations that received an increasing number of migrants from the Tokyo Metropolis or the Tokyo special wards (Fielding & Ishikawa, 2021; Kotsubo & Nakaya, 2022). In addition to the increase of keen interest in residential relocation to provincial areas in TMA reported by the Cabinet Office (2021), Teng (2022) reported that young adults’ working environment, income and psychological status were significantly worsened by the COVID‐19 pandemic and that these changes were related to their migration intentions from Tokyo. In addition, Tsuboi et al. (2021) pointed out changes in residential preference from before to after the COVID‐19 pandemic, such as increased interest in residing in the suburbs, in the case of Toyota city, a provincial city.

Overall, the most significant impact of the pandemic was in the TMA. The changes in migration intentions and residential preferences may lead to an increase in out‐migrants from the centres of cities, including the TMA, and to a decrease in in‐migrants to there. In addition, we would expect an increase in migration from the centre to the suburbs within a city. Based on the increase in net migration to rural areas in other countries during the pandemic (González‐Leonardo et al., 2022; Stawarz et al., 2022), rural areas are also expected to have been destinations of migrants from cities in the case of Japan.

To capture changes in internal migration patterns at the above geographical scales (e.g., core–peripheral and centre–suburbs distinctions), we use intermunicipal migration flows rather than interprefectural ones as used in previous studies.

## 3 METHODS

### 3.1 Iterative Proportional Fitting

We use iterative proportional fitting (IPF) to estimate the suppressed cells in the OD matrices of Japanese intermunicipal migration data. IPF is a mathematical scaling procedure used to ensure that a two‐dimensional table of data is adjusted such that its row and column totals agree with the constraining row and column totals (Norman, 1999). It is well‐known that the results of estimation are a means to entropy‐maximising based on the maximum‐likelihood estimation (Johnston & Pattie, 1993). IPF is particularly suited to estimate migration flows as the data sets are often available for total moves into and out of an area, but the data for the interaction between these areas are often not available (Lomax & Norman, 2016). There are many applications of IPF in migration studies (e.g., Chilton & Poet, 1973; Lomax et al., 2013; Rees & Duke‐Williams, 1997).

In the IPF procedure, the cell values in a table are recalculated so that the row and column sums of the cells become equal to the marginal totals. Following previous studies (Lomax et al., 2013; Norman, 1999; Wong, 1992), IPF procedure is described as iterations of the following two equations:

(1) $$p_{\mathrm{ij}(k+1)}=\frac{p_{\mathrm{ij}(k)}}{\sum_{j}p_{\mathrm{ij}(k)}}\times Q_{i},$$

(2) $$p_{\mathrm{ij}(k+2)}=\frac{p_{\mathrm{ij}(k+1)}}{\sum_{i}p_{\mathrm{ij}(k+1)}}\times Q_{j},$$

where $p_{\mathrm{ij}(k)}$ is the matrix element in row i, column j and iteration k. $Q_{i}$ and $Q_{j}$ are the predefined row totals and column totals, respectively. Theoretically, the iterative estimation process will stop at iteration m when

(3) $$\sum_{j}p_{\mathrm{ij}(m)}=Q_{i}\text{and}\sum_{i}p_{\mathrm{ij}(m)}=Q_{j}.$$

Instead, this study uses the following stopping rule:

(4) $$\left|p_{\mathrm{ij}(k+2)}-p_{\mathrm{ij}(k+1)}\right|<\beta \text{for all}p_{\mathrm{ij}},$$

where β is the threshold value to stop the iteration.

### 3.2 Data and preparations for IPF

**Figure 1:**
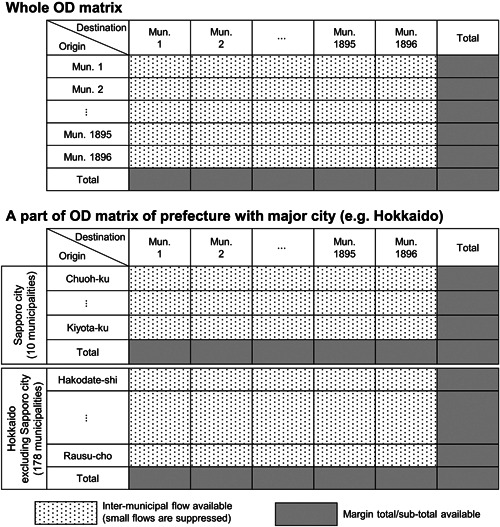
Data availability of annual intermunicipal migration in Japan. OD, origin–destination.

The data source for the annual intermunicipal migration since 2012 is the Annual Report on the Internal Migration in Japan derived from the Basic Resident Registers published by the Statistics Bureau in the Ministry of Internal Affairs and Communications. The data are collected based on calendar year; for instance, ‘2020’ refers to the period from 1 January to 31 December 2020. Figure 1 illustrates the available data in a matrix form.

The annual report publishes not only OD matrices of intermunicipal migration but also the total number of in‐migrants from other municipalities, total out‐migrants to other municipalities and total in‐migrants of each municipality by prefecture and by each of the 21 major cities (Tokyo special wards and 20 of the government‐ordinance‐designated cities). For example, the OD matrix with municipalities of Hokkaido as the origins can be divided into two parts with available row and column totals: municipalities of Sapporo city, which is one of the government‐ordinance‐designated cities, and the rest of the municipalities, are as shown in Figure 1.

These data cover Japanese migrants during the period 2012–2017; since 2018, they also include other nationalities. The OD matrices and numbers of in‐migrants and out‐migrants are separately available for Japanese people and other nationalities since 2018. The proportions of non‐Japanese migrants to the total were 8.8%, 9.5%, and 7.9% in 2018, 2019 and 2020, respectively. While the coverage of in‐migrants by prefecture and by each of the 21 major cities was only for Japanese migrants from 2012 to 2017, the coverage changed to include Japanese and all nationalities from 2018; however, these data by nationality are not published.

Although there are some suppressed cells in the OD matrix, the numbers of in‐migrants and out‐migrants without suppression are available. The data source states that small flows are suppressed in the OD matrices but do not provide the threshold of suppression. The proportion of suppressed migrants was 23.3% in 2020, and the level has remained almost the same during the study period (as shown in Table A2).

Using the number of in‐migrants and out‐migrants as the marginal subtotal and total, the OD matrix can be divided into 68 regions (21 major cities and 47 prefectures, excluding these cities) by row directions with row and column totals. We apply IPF to each divided OD matrix to estimate suppressed cells, making the best use of available data. Due to the division of OD matrices using the marginal totals, the post‐2018 data cannot be adjusted to give data just for the Japanese. Hence, the results in this paper have the limitation of discontinuity in the time series between 2017 and 2018.

To estimate the suppressed cells in the OD matrices using IPF, four steps of preprocessing the data were conducted:

(1) The difference between the number of out‐migrants of each municipality and the sum of migrants from i in the OD matrix was calculated to determine the predefined row totals $Q_{i}$.
(2) The difference between the number of in‐migrants of each municipality and the sum of migrants to j in the OD matrix was calculated to determine the predefined column totals $Q_{j}$.
(3) The known cells and intramunicipal ones were replaced by 0 and the others were replaced by 1 as the initial values of $p_{\mathrm{ij}}$.
(4) Rows with $Q_{i}=0$ and columns with $Q_{j}=0$ were removed because they indicate that the number of migrants is zero in the suppressed cells.

Through these preprocessing steps, the suppressed cells are classified into three types: the cells substituted by 1, removed from the OD table and intramunicipal flows substituted by 0. The removed cells can be regarded as those of no migrants by the constraints of row and column totals. We conduct the IPF estimation of suppressed cells as $p_{\mathrm{ij}(0)}=1$ using the OD matrix, $Q_{i}$ and $Q_{j}$ from the above preparation 68 times each year. Then we obtain the corrected OD matrices, including the estimated migrants of the originally suppressed cells. We set the threshold value β in Equation 4 as 0.0001.

### 3.3 Area classification

**Figure 2:**
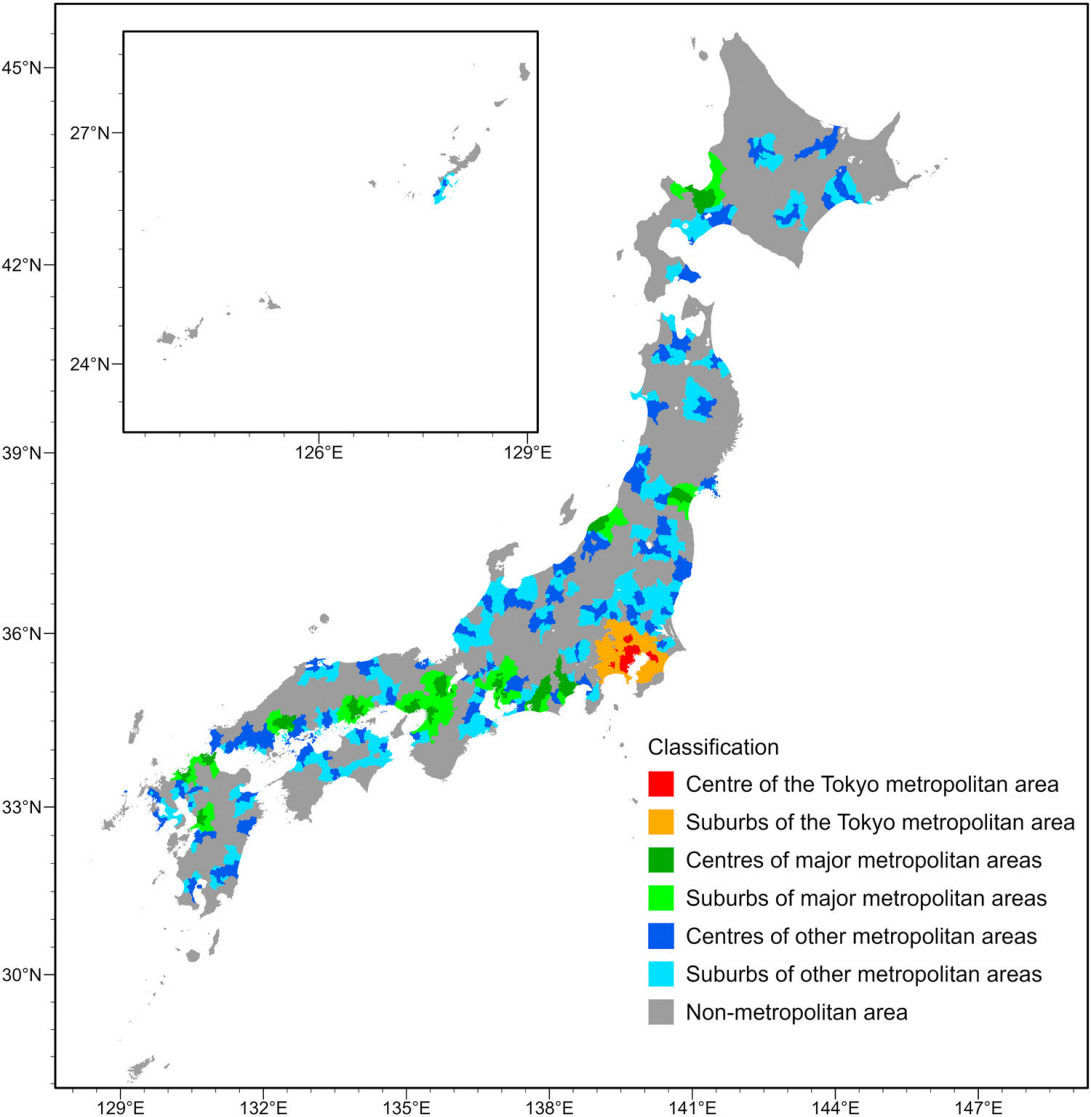
Classification of Japanese municipalities

Area classifications are useful to summarise the macrotrends in migration studies (e.g., Ambinakudige & Parisi, 2017; Lomax et al., 2014). Previous studies have discussed internal migration in Japan based on several kinds of dichotomy between core and periphery, like TMA versus other regions (Ishikawa & Fielding, 1998; Tabuchi, 1988), major metropolitan areas versus minor/peripheral metropolitan areas (Morikawa, 2006) and city centre versus commuting suburbs in a metropolitan area (Koike, 2017). Following the definition of Urban Employment Area (Kanemoto & Tokuoka, 2002) using the commuting network of the 2015 Census, we identify three types of metropolitan areas: the Tokyo metropolitan area (TMA), major metropolitan areas (MMAs), which contain government‐ordinance‐designated cities as their centre, and other metropolitan areas (OMAs). In each metropolitan area, a centre is composed of densely populated municipalities, and a suburb is an area where at least 10% of workers commute to the centre (for further details, see Appendix B). Following the distinction between centre and suburb, we then classify municipalities into seven types: centre of the TMA, suburbs of the TMA, centres of MMAs, suburbs of MMAs, centres of OMAs, suburbs of OMAs, and nonmetropolitan area. Figure 2 shows the classification of municipalities.

In this study, we aggregate the intermunicipal migration flows into the interregional flows among the seven area classifications to summarise the macro trends in internal migration in Japan.

### 3.4 Measuring temporal changes in internal migration

To understand the trends and changes in internal migration, net migration rates are commonly used. However, the net migration rate is based on information about migration and regional population, making it a function of the differential propensities of its components (Lieberson, 1980). Hence, the results are affected by the cumulative population history (Plane, 1984; Stillwell et al., 2000). To overcome this shortcoming, a family of ratios, known collectively as the migration effectiveness index, has been proposed (Stillwell et al., 2000). This family of ratios is the net migration divided by its constituent flows; it measures the efficiency of population redistribution. These ratios are superior to the conventional net migration rate for a systematic analysis of internal migration patterns (Plane, 1984). In this study, we use net migration to measure the size of migration and migration efficiency ratios (MERs) to measure temporal changes in internal migration patterns.

We first calculate the MER by the seven area types of municipal classification as

(5) $${\mathrm{MER}}_{a}=100\times \frac{D_{a}-O_{a}}{D_{a}+O_{a}},$$

where ${\mathrm{MER}}_{a}$ is the MER of area type a, $D_{a}$ is the total inflows from other areas to a and $O_{a}$ is the total outflows from a to all other areas. This index varies from −100 to 100; a large absolute value of the ratio indicates unidirectional migration flows and the value 0 indicates that in‐migrations and out‐migrations are equal. When the index is positive, it indicates a population gain of a and a negative index represents a population loss.

In addition, we compute the stream‐specific MER as

(6) $${\mathrm{MER}}_{\mathrm{ab}}=100\times \frac{M_{\mathrm{ab}}-M_{\mathrm{ba}}}{M_{\mathrm{ab}}+M_{\mathrm{ba}}},$$

where ${\mathrm{MER}}_{\mathrm{ab}}$ is the stream‐specific MER based on the stream from area type a to area type b, and $M_{\mathrm{ab}}$ is the migration stream from a to b. The stream‐specific MER takes a value from −100 to 100. The positive sign indicates a net gain of b from migration flows and the negative sign indicates a net loss. The sign of the stream‐specific MERs is opposite that of its counter stream. A large absolute value of the ratio can be interpreted as a large net effect on population redistribution for a given volume of the stream.

## 4 RESULTS

### 4.1 Net migration patterns in Japan

**Figure 3:**
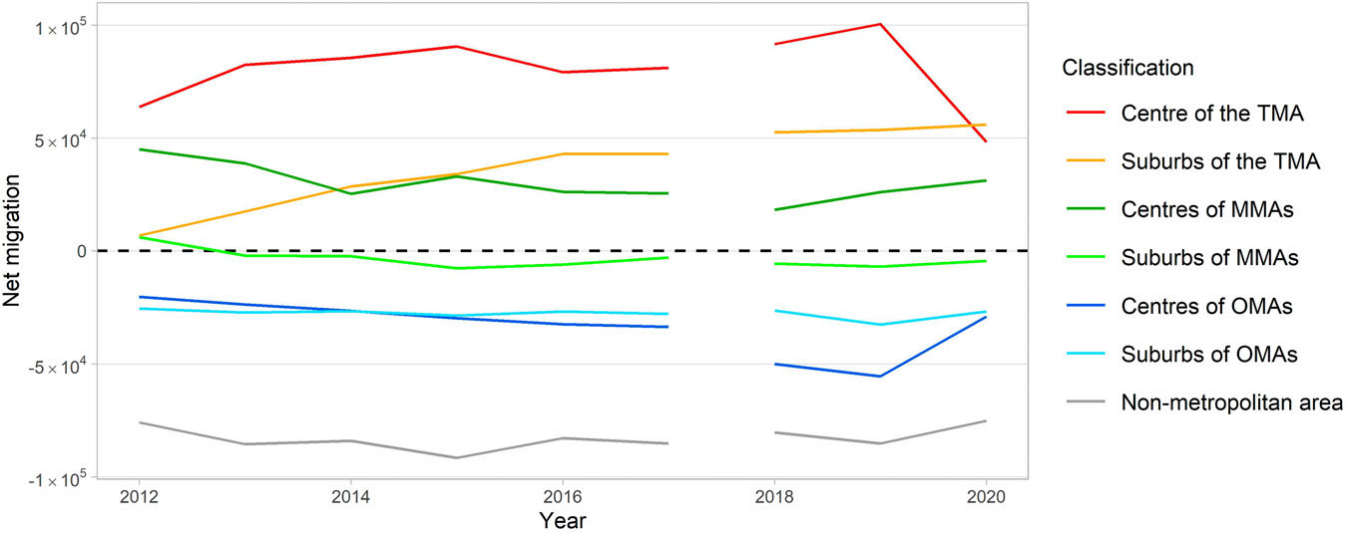
Net migration from 2012 to 2020. The data cover Japanese until 2017 and Japanese and other nationalities since 2018. MMA, major metropolitan area; OMA, other metropolitan area; TMA, Tokyo metropolitan area.

Figure 3 shows trends in net migration patterns by the area of classification.

The TMA has retained the net gain from other areas over the entire study period. The size of the net migration to the centre of the TMA remained steady, but had approximately 90,000 and 100,000 as the peaks in 2015 and 2019, respectively. Additionally, the net gain of the suburb of the TMA also showed an increase. These trends are in line with findings from previous studies (Fielding, 2018; Ishikawa, 2020b). The centre of the TMA showed a mass net gain, while the suburbs of the TMA and the centre of MMAs showed relatively lesser gains. However, the suburbs of MMAs had almost zero or zero net loss, while a large net loss was observed in the other metropolitan and nonmetropolitan areas. This net migration trend persisted from 2012 to 2019. In 2020, the net gain of the suburbs of the TMA was larger than that of its centre.

**Figure 4:**
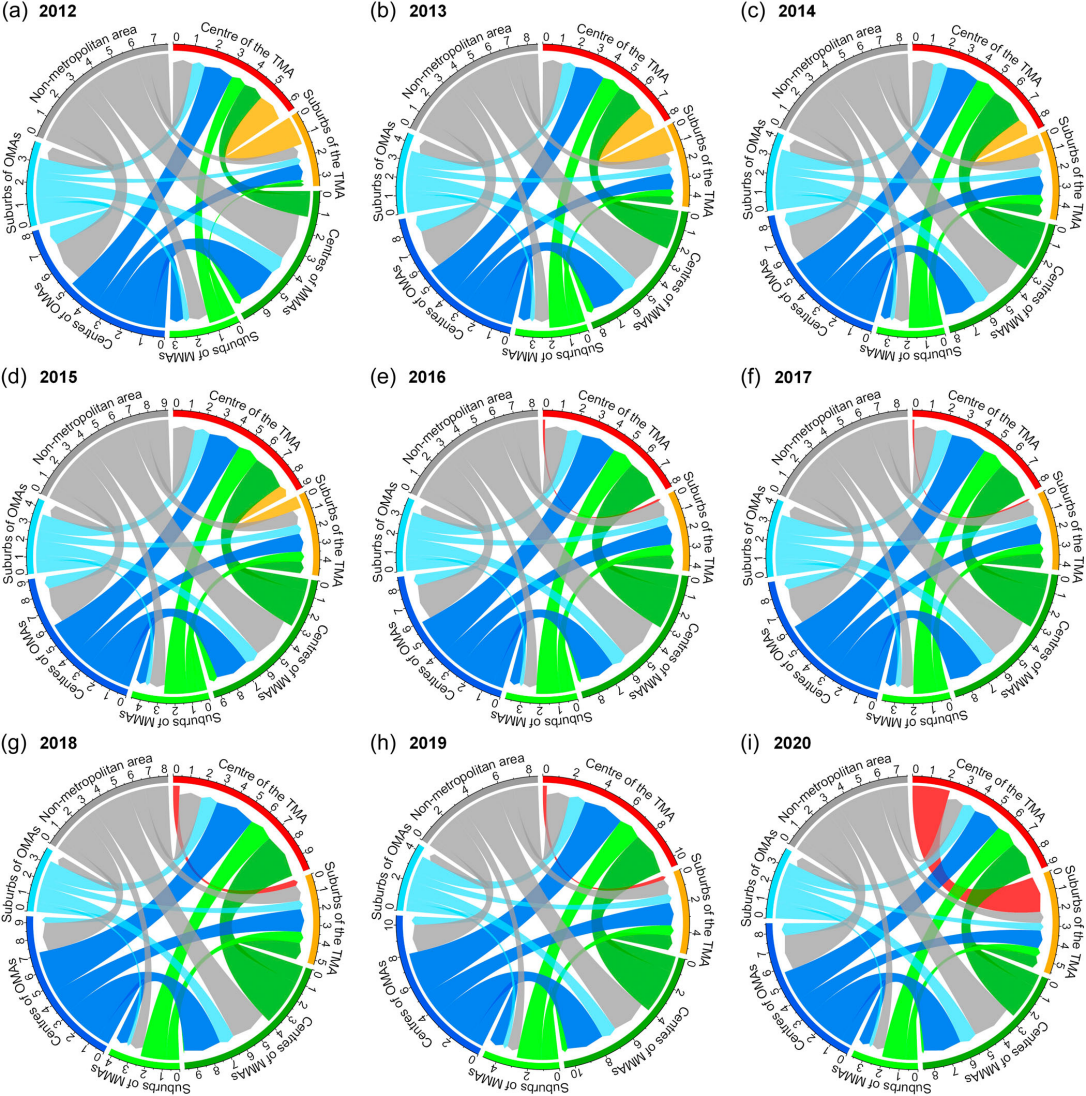
Circular plots of estimated net migration flows among seven areas. (a)–(i) Net migration flows from 2012 to 2020. Numbers on the outer section axis represent the size of migration flows in 10^4^ individuals. The data cover Japanese migrants until 2017 and Japanese and other nationalities since 2018.

**Figure A1:**
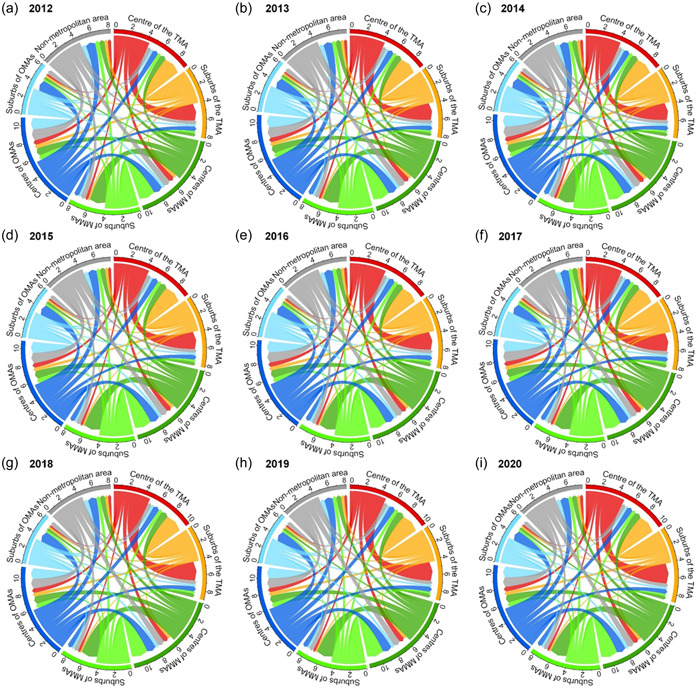
Circular plots of estimated internal migration flows in Japan among seven areas. (a)‐(i) migration flows in 2012–2020. Note: Numbers on the outer section axis represent the size of migration flows in 10^5^ of individuals. The data cover Japanese until 2017 and Japanese and other nationalities since 2018.

Figure 4 represents the estimated net migration flows aggregated by the area classification (Figure A1 shows internal migration flows among the areas).

Focusing on the direction of migration flows, the centre of the TMA has net gains from all other areas from 2012 to 2015. However, the flow from the centre to the suburbs within the TMA became slightly larger than its counter in 2016, and this condition continued until 2019. In 2020, the size of the net flow increased from approximately 3000 in 2019 to 20,000. The MMAs had net gains from OMAs and nonmetropolitan areas and OMAs did from nonmetropolitan areas. The sizes of flows from centres to suburbs of MMAs and OMAs were almost the same as those of their counters in the study period and did not change significantly in 2020, like the case of the TMA.

### 4.2 Migration efficiency based on intermunicipal flows

**Figure 5:**
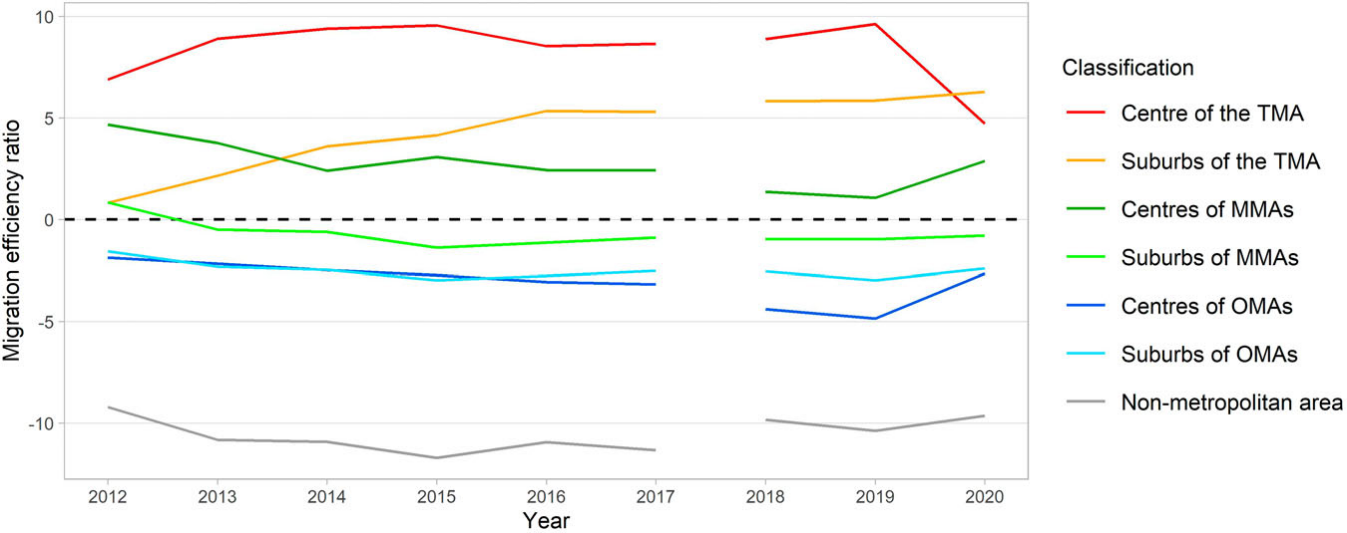
Migration efficiency ratio from 2012 to 2020. The data cover Japanese until 2017 and Japanese and other nationalities since 2018. MMA, major metropolitan area; OMA, other metropolitan area; TMA, Tokyo metropolitan area.

Figure 5 shows the changes in MER from 2012 to 2020 by the seven types of area classification based on the estimated data.

Trends of MER were almost the same as those of net migration, as shown in Figure 4. Focusing on the sign of the MER, the centre and suburbs of the TMA and the centre of MMAs showed net gains, while the other areas showed a net loss in the period. The value of the ratio in the centre of the TMA remained steady, but an upward trend was seen in the ratio in the suburb of the TMA between 2012 and 2017. Considering the increase in the size of net migration in the suburbs of the TMA, as shown in Figure 3, this might suggest that the attractiveness of the suburb increased in the study period, and the contribution of net migration in the suburbs became strong with the recent population concentration in the TMA.

While the value of ratios in areas other than TMA decreased or levelled off, a significant change in 2020 led to a sharp drop in the ratio in the centre of the TMA, while the ratios in all other areas increased. These indicate that, despite the net gain, the population concentration of the TMA became weak. It is notable that the MERs of the centre of MMAs and OMAs showed a huge increase in 2020, implying that the centre of urban areas, excluding the TMA, received relatively large benefits in terms of population concentration during the COVID‐19 pandemic rather than the other areas.

Although precise comparisons of the values in the whole study period cannot be conducted due to the difference in data coverage, the value of the MER of the centre of the TMA in 2020 is the lowest in the study period, from 2012. While there is a weakening of population concentration, the values of MERs of the centre and suburbs of the TMA were positive, and the TMA continued to receive net population gain. Therefore, the pandemic did not alter the existing structures of the national migration system: population losses in provincial areas and gains in large cities.

**Figure 6:**
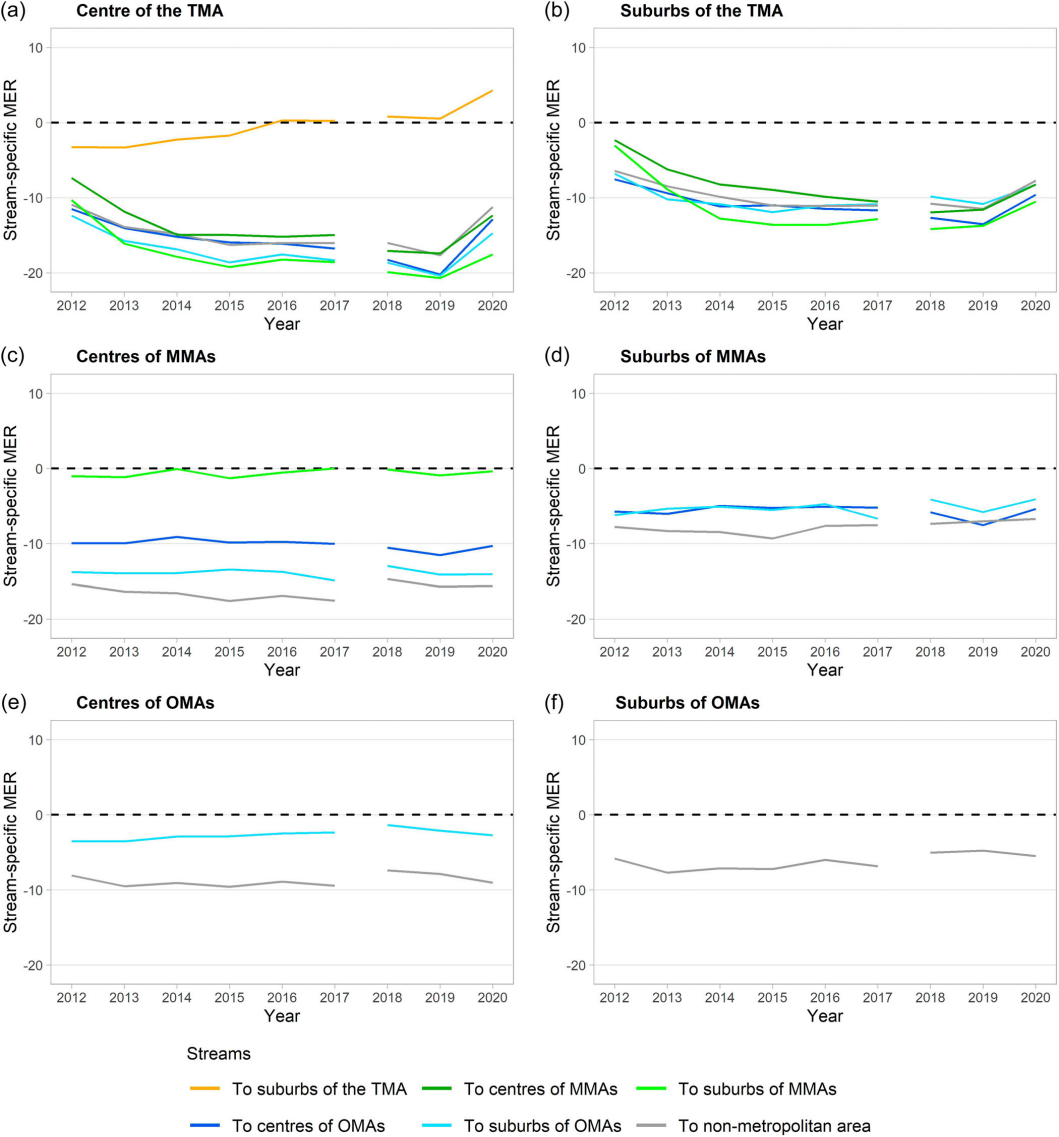
Stream‐specific migration efficiency ratio from 2012 to 2020. (a) Streams from centre of the TMA, (b) streams from suburbs of the TMA, (c) streams from centres of MMAs, (d) streams from suburbs of MMAs, (e) streams from centres of OMAs, and (f) stream from suburbs of OMAs. The data cover Japanese until 2017 and Japanese and other nationalities since 2018. MER, migration efficiency ratio; MMA, major metropolitan area; OMA, other metropolitan area; TMA, Tokyo metropolitan area.

Figure 6 shows the stream‐specific MER from 2012 to 2020 by a combination of the seven area types of classification based on the estimated data.

Focusing on the sign of the ratios, the centre and suburbs of the TMA showed net gains when compared to other areas during the period, as shown in Figure 6a,b. The values of these showed a tendency to decrease by 2019, but they increased in 2020. There were significant changes in the ratios based on the stream from the centre of the TMA to the centre of MMAs and OMAs and nonmetropolitan areas. Regarding the stream within the TMA, the ratio based on the stream from its centre to suburbs was close to zero, but changed from negative to positive in 2016. Additionally, there was a relatively large increase in the ratio in 2020.

In the case of the centres and suburbs of MMAs, the MERs based on the stream to OMAs and nonmetropolitan areas showed negative values as shown in Figure 6c,d. The value of the ratio based on the stream from the centres to the suburbs of MMAs remained almost zero. While the MERs based on the stream from the centre of MMAs to the centre of OMAs and that from the suburbs of MMAs to the centres and suburbs of OMAs increased in 2020, the values were almost the same as those in 2018. Hence, all the ratios apparently levelled off in the period.

The MERs based on the streams from the centre and suburbs of OMAs to nonmetropolitan areas had negative values, as shown in Figure 6e,f in the study period. The ratio based on the stream from the centre to the suburb of others also showed negative values. Similar to the case of MMAs, all ratios remained at a certain level during the study period.

Regarding changes in 2020, the MER based on the stream from the centre of the TMA to its suburb increased, indicating that the pandemic accelerated migration from the centre to the suburb, showing an increase from previous years. By contrast, the stream‐specific MERs unrelated to TMA remained the same as that before 2020. Accordingly, these changes reveal that the COVID‐19 pandemic strongly affected migration patterns to/from and within the TMA while the impact was minimal for migration among the other areas.

## 5 DISCUSSION

### 5.1 Trends in internal migration to and within the TMA in 2012–2019

A recent trend is a large‐sized internal migration in Japan from rural to urban areas, which can be observed as net migration, as shown in Figures 3 and 4, with the signs and values of MER as shown in Figure 5. This trend is particularly represented by the population concentration of the TMA, as previous studies have pointed out (Fielding, 2018; Ishikawa, 2020b). The MERs of the centre and suburbs of the TMA increased from 2012 to 2017, as seen in Figure 5, suggesting a sharp population concentration in the TMA during the period. The increase of MER in the suburb of TMA was especially large, from almost 0% in 2012 to 5% in 2017. Besides, it is noteworthy that the MER based on the stream from the centre of the TMA to its suburbs changed from a negative value to a positive one, as shown in Figure 6. This is in contrast to the population returning to the central parts of cities since the 1990s, as pointed out by a previous study (Inoue et al., 2022), and is a finding not seen in previous studies using prefectural‐scale data.

Accordingly, the results in 2012–2019 showed a strengthening of population concentration in urban areas. The economic boom in Japan during this period was considered a recovery after the global economic crisis of 2008 and the Great East Japan Earthquake and Tsunami of 2011. For instance, the unemployment rate decreased, and the active opening ratio, which is defined as the number of active job openings divided by the number of active applicants, increased (Japan Institute for Labour Policy and Training, 2021). Since a positive relationship exists between migration to the metropolitan area and business cycles in Japan (Fielding, 2018; Ishikawa, 2020b), the upturn of the economy affected the population concentration in urban areas from 2012 to 2019.

The results indicate that migration from the centre to the suburbs within the TMA gradually increased compared to its counter. Moreover, the stream‐specific MERs based on the streams from the suburbs of the TMA to other areas decreased. In developing the suburbs of the TMA, the suburbanisation of office location progressed rapidly, thus enhancing the polycentricity of the TMA (Yamamura & Goto, 2010). Therefore, the changes in the indices related to the suburbs of the TMA might capture the structural changes in the TMA with the increasing attractiveness of its suburbs as workplaces. However, there were salient changes in two indices in 2020 when the COVID‐19 pandemic spread.

### 5.2 Impact of COVID‐19 in 2020

In 2020, the MER of the centre of the TMA showed the lowest value in the study period. In addition, the values of stream‐specific MERs from the TMA to other area types greatly increased, indicating that the impact of the COVID‐19 pandemic was largest on the TMA among the seven area types. Considering the situational changes caused by the COVID‐19 pandemic, the decrease of MER of the TMA would reflect an increase in its propulsiveness as well as a decrease in its attractiveness as a place to live, especially in the centre of the TMA.

Accordingly, the COVID‐19 pandemic accelerated the traditional movement patterns from the centre to the suburbs within the TMA (Watanabe, 1978), indicating a new urban sprawl threat (Tsuboi et al., 2021). While the MER of the suburb of the TMA increased, the stream‐specific MERs indicate that the suburbs received much net gain from their centre. It has been noted that shopping, eating out, hobbies, and recreational activities increased around the home among urban residents compared to the same before the pandemic (Ministry of Land Infrastructure Transport and Tourism, 2022). This suggests an increase in the importance of the home and its neighbourhood environment. A previous study has pointed out that teleworking weakened the restrictions on residence and place of employment (Haslag & Weagley, 2021). The moves from the centre to the suburb within the TMA may have been partially caused by the locational preference of teleworkers (de Abreu, 2022; Denham, 2021), with an anticipation of postpandemic hybrid work, such as commuting few times a week to the centre. This indicates that access to the workplace will remain an important factor for the movers.

There were large increases in MERs of the centres of MMAs and OMAs and stream‐specific MERs from the centre of the TMA to the centres of MMAs and OMAs in 2020. These centres were known as ‘pumps’ or ‘siphon cities’, which attract people from their surrounding regions and then channel them to the TMA for employment and career development (Fielding & Ishikawa, 2021; Morikawa, 2020). Thus, the COVID‐19 pandemic reduced out‐migration to the TMA and increased in‐migration from the TMA in a tendency to avoid the extra dense‐populated areas in the TMA. On the contrary, the stream‐specific MERs unrelated to the TMA showed the same trends in migration from small metropolitan areas to large ones and from suburbs to the centre within a metropolitan area. The tendency to avoid densely populated city centres in non‐TMA regions was seemingly weak compared to the case of the TMA.

While all MERs excluding that of the centre of the TMA increased in 2020, the value of the MERs of nonmetropolitan areas remained negative and almost the same as that in 2018 and 2019. In contrast, in countries like Spain and Germany, net migration greatly increased in rural areas (González‐Leonardo et al., 2022; Stawarz et al., 2022). Some of the characteristics of findings from previous studies on Japan similar to the studies on Spain were that rural destinations are relatively easily accessible from cities; for instance, places with rich natural resources and scenic beauty are not far from Tokyo or Madrid: the mountain villages neighbouring Madrid in Spain (González‐Leonardo et al., 2022) and small towns in Nagano Prefecture, which are tourist destinations, are only a few hours away from Madrid and Tokyo, respectively (Fielding & Ishikawa, 2021; Kotsubo & Nakaya, 2022). Thus, a few rural municipalities in Japan may have experienced a sudden increase in the number of in‐migrants during the pandemic depending on their natural environment and their accessibility to Tokyo. However, the negligible change of MER of the entire nonmetropolitan area indicates that the impact of the pandemic was small compared to the cases of rural areas in Spain and Germany.

Although there were significant changes in the trends in internal migration in Japan due to the pandemic, as mentioned above, it is necessary to examine whether these changes persisted after the pandemic. It is noteworthy that while the pandemic weakened the population concentration in the TMA, the region continues to receive net gains in 2020. The factors of population concentration such as regional differences in employment opportunities and income (Ishikawa, 2020b; Takami, 2018) continue to exist regardless of the pandemic. If the economy improves after the pandemic, the population concentration in Tokyo is expected to strengthen, similar to the trend observed in the period 2012–2019, with a positive relationship between migration to metropolitan areas and economic boom (Fielding, 2018; Ishikawa, 2020b). The population retention within the TMA and its continued net gain indicate the importance of the TMA as a place for upward socio‐occupational mobility (Fielding, 2018; S. Nakagawa, 2000) even in the midst of the pandemic. Hence, reducing regional differences is critical in rectifying the ‘monopolar concentration’ into Tokyo in the long term after the pandemic.

Additionally, the changes in migration preferences related to teleworking caused by the pandemic serve as a political opportunity to promote building digital infrastructure, such as the ‘Vision for a Digital Garden City Nation’, which aims to reduce regional differences between urban and rural areas (Government of Japan, 2022). It may enable wider adoption of teleworking to promote migration from urban to rural areas without a job change, as well as create new working opportunities in rural areas. However, our results show that the impact of the pandemic in nonmetropolitan areas is not prominent. While restrictions between home and workplaces are weakened by teleworking, our results show that maintaining accessibility to the centre of the TMA is still an important factor while choosing a residential location. Therefore, concerns about the development of urban sprawl within the TMA prevail owing to the wide adoption of teleworking, which has led to increased movement from its centre to its suburbs rather than to other areas. This phenomenon has the potential for growing fiscal pressure on maintaining infrastructures in Japan, where the total population is on the decrease. It will therefore be necessary to keep a close watch on future trends in internal migration in Japan in conjunction with social, economic and political aspects.

## 6 CONCLUSIONS

In this study, we examined the internal migration trends from 2012 to 2020 in Japan. The estimated intermunicipal migration flows were aggregated based on seven area classifications, with distinctions of core–periphery and centre–suburbs of metropolitan areas, to examine migration trends not seen in previous studies using prefectural‐scale data. From 2012 to 2019, the change in the indices showed a migration trend towards urban areas, especially the TMA. Within the TMA, the trend gradually changed as migration from the centre to suburb became large compared to its counter. However, in 2020, the trend changed drastically with the outbreak of the COVID‐19 pandemic. The changes in the indices demonstrate that the pandemic certainly weakened the population concentration within the TMA. Although the impact was relatively large in the centre of the TMA, net gains were observed both in the centre and the suburb. This is an indication that the impact of the pandemic was small in rural areas, in contrast to the case of rural areas in Spain and Germany (González‐Leonardo et al., 2022; Stawarz et al., 2022). The results also indicate that the pandemic strengthened the migration from the centre to the suburb within the TMA, showing a gradual increase, which indicates a renewed threat of urban sprawl. These changes can be observed using relatively fine time and geographical scale data owing to the upgrade of the data source and our estimation. Furthermore, these findings can help with the postpandemic policy challenge outlined in a prior study (Denham, 2021), which needs to consider how experiences of and responses to the pandemic may be harnessed to foster economic development and employment growth in nonmetropolitan regions based on past migration patterns.

There are some limitations in this study. First, our results use a discontinuous time series of migration trends due to data coverage limitations. The change of coverage might affect the results, especially in the TMA and MMAs, because foreign migrants have relatively high mobility and a tendency to move to large cities compared to the Japanese (M. Nakagawa et al., 2016). However, because the proportions of non‐Japanese migrants to the totals in 2018–2020, were lower than 10%, we would assume the change of data coverage was not a severe problem. On the one hand, we estimated migration flows by 68 parts of the OD matrix, to make the best use of available data. On the other hand, it might be possible to estimate intermunicipal Japanese migration flows based on the whole matrix, albeit with relatively poor accuracy. Considering the possibility that foreign residents and their share of migrants may increase, it will be necessary to estimate harmonised time series for comparison of long‐term trends in future work. Second, to clarify the determinant factors of recent migration patterns in Japan, additional empirical analysis is required. Considering the population concentration of the TMA, it is important from a policy perspective to grasp the effects of some regional differences such as income, industrial structure, and land prices on in‐migration and out‐migration (Ishikawa & Fielding, 1998; Tabuchi, 1988; Tanaka, 2017). Additionally, the occupational structure, such as the share of knowledge workers, is an important factor while considering future trends in internal migration during and after the COVID‐19 pandemic (Denham, 2021). However, the volume of migration flow generally depends on the factor of origin and destination and their relationship (Lee, 1966; Ullman, 1980). To make the best of the estimated OD data, analysis considering the relationships between municipalities needs to be undertaken in future work. Finally, the estimation process is simple; however, the results are not in integer form. Future studies will need to seek some proper rounding procedures and alternative estimation methods that may improve the estimation.

## CONFLICT OF INTEREST

The authors declare no conflict of interest.

## Data Availability

The data before the estimation are publicly available from e‐Stat (https://www.e-stat.go.jp/en), which is a portal site for Japanese Government Statistics. The definition of Urban Employment Area, which is the base of area classification in this study, is available online (https://www.csis.u-tokyo.ac.jp/UEA/index_e.htm).

## APPENDIX A

**Table A1:** List of abbreviations and acronyms

Abbreviation/acronym	Definition
IPF	Iterative proportional fitting
MER	Migration efficiency ratio
MMA	Major metropolitan area
OD	Origin‐destination
OMA	Other metropolitan area
TMA	Tokyo metropolitan area

**Table A2:** Proportion of suppression of migrants

Year	Total migrants published	Total migrants in OD matrix	Proportion of suppressed migrants (%)
2012	5,018,166	3,816,794	23.9
2013	5,015,572	3,812,222	24.0
2014	4,908,009	3,720,366	24.2
2015	5,041,483	3,837,855	23.9
2016	4,880,966	3,692,152	24.4
2017	4,893,580	3,709,975	24.1
2018	5,359,174	4,120,785	23.1
2019	5,403,464	4,155,136	23.1
2020	5,255,721	4,029,812	23.3

*Note*: The data cover Japanese migrants until 2017 and Japanese and other nationalities since 2018.

## APPENDIX B

### Urban Employment Area

Urban Employment Area is one of the boundaries based on employment patterns proposed by Kanemoto and Tokuoka (2002). The rules to establish urban employment area are following: (1) Municipalities which have densely inhabited districts with population above 50,000 or more are classified into centres of urban employment area. A densely inhabited district is defined as more than 4,000 people/km^2^ per measuring a census basic unit and more than 5,000 people including the neighbouring the units in a municipality. (2) Municipalities where at least 10% of workers commute to a specific centre are identified as suburbs of the centre. (3) Multiple centres exist within the same area if sets of municipalities comprising centres and suburbs follow the above rules.
